# Long Non-coding RNAs as Promising Therapeutic Approach in Ischemic Stroke: a Comprehensive Review

**DOI:** 10.1007/s12035-020-02206-8

**Published:** 2020-11-24

**Authors:** Marta Wolska, Joanna Jarosz-Popek, Eva Junger, Zofia Wicik, Tahmina Porshoor, Lucia Sharif, Pamela Czajka, Marek Postula, Dagmara Mirowska-Guzel, Anna Czlonkowska, Ceren Eyileten

**Affiliations:** 1 Department of Experimental and Clinical Pharmacology, Medical University of Warsaw, Center for Preclinical Research and Technology CEPT, Banacha 1B str., Warsaw, 02-097 Warsaw, Poland; 2 Centro de Matemática, Computação e Cognição, Universidade Federal do ABC, Sao Paulo, Brazil; 3 2nd Department of Neurology, Institute of Psychiatry and Neurology, 02-957 Warsaw, Poland

**Keywords:** Non-coding RNA, lncRNA, Stroke, Treatment, Ischemia/reperfusion, Therapeutic approach, Novel therapy

## Abstract

In recent years, ischemic stroke (IS) has been one of the major causes of disability and mortality worldwide. The general mechanism of IS is based on reduced blood supply to neuronal tissue, resulting in neuronal cell damage by various pathological reactions. One of the main techniques for acute IS treatment entails advanced surgical approaches for restoration of cerebral blood supply but this is often associated with secondary brain injury, also known as ischemic reperfusion injury (I/R injury). Many researches have come to emphasize the significant role of long non-coding RNAs (lncRNAs) in IS, especially in I/R injury and their potential as therapeutic approaches. LncRNAs are non-protein transcripts that are able to regulate cellular processes and gene expression. Further, lncRNAs have been shown to be involved in neuronal signaling pathways. Several lncRNAs are recognized as key factors in the physiological and pathological processes of IS. In this review, we discuss the role of lncRNAs in neuronal injury mechanisms and their association with brain neuroprotection. Moreover, we identify the lncRNAs that show the greatest potential as novel therapeutic approaches in IS, which therefore merit further investigation in preclinical research.

**Figure:**
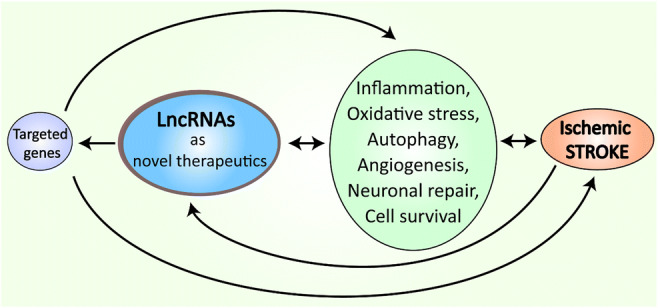
Graphical Abstract

Graphical Abstract

## Introduction

Ischemic stroke (IS), which constitutes more than 80% of strokes, generally results from reduced blood supply to cerebral tissue resulting in a number of pathological reactions such as oxidative stress, inflammation, and neuronal cell death [1]. Since IS is one of the major causes of disability worldwide, and since pharmacological approaches have generally failed in translation, there is a genuine requirement for novel therapeutic approaches. Previous researches have focused on preventing IS among those at risk, reducing severity, and promoting neurogenesis and neuronal recovery after IS. Early restoration of cerebral blood perfusion in ischemic tissues plays a pivotal role in IS treatment in order to minimize the severity of brain injury and neurological impairment [2]. Nevertheless, IS often leads to a secondary brain injury called ischemic/reperfusion (I/R) injury. The precise underlying mechanism of this injury remains unknown; however, many researchers have come to emphasize the role of inflammation, autophagy, and apoptosis as important contributors [3]. Moreover, vascular neural networks, especially brain microvascular endothelial cells (BMECs), are also prone to reperfusion damage. The I/R injury of BMECs leads to blood-brain barrier disruption, furthers brain injury, and is associated with poor prognosis in IS patients [4]**.**

Long non-coding RNAs (lncRNAs) are a class of non-protein transcripts which are greater than 200 nucleotides in length. LncRNAs play a crucial role in various cellular processes, including differentiation, apoptosis, as well as regulation of genes expression [2, 5]. LncRNA alterations are often associated with a dysregulation of signaling pathways that control multiple neuronal, physiological, and pathophysiological processes. Additionally, lncRNAs can act as competing endogenous RNAs (ceRNA) of microRNAs (miRNAs) by binding to them, thereby reducing their regulatory effects on mRNA. MiRNA-mRNA interactions play an important role in the pathogenesis, diagnosis, and treatment of several diseases, including cardiovascular diseases, diabetes, as well as IS [1, 6–10]. LncRNAs, due to their broad range of action, can be targeted by various approaches either in the nucleus or in the cytoplasm. Firstly, small interfering RNA (siRNA), which activates the RNA-induced silencing complex, allows for post transcriptional RNA degradation. A similar degradation effect but with a different mechanism is achieved by antisense oligonucleotides. Besides, steric blockage of the promoter as well as techniques affecting the genome allows for lncRNAs gene regulation. RNA binding small molecules or morpholino oligonucleotides allow for RNA-protein interaction inhibition [11]. Nevertheless, there are many limitations associated with targeting lncRNAs as therapeutics in diseases, including crossing cellular plasma membrane and immune system response to foreign RNAs, resulting in a limited number of studies in this area.

In this review, we focus on lncRNAs that are able to regulate key factors involved in I/R injury such as calcium overload or glutamate toxicity. We present the relationship between lncRNAs and pathological processes that can contribute to ischemic injury exacerbations such as autophagy, inflammation, and oxidative stress. Moreover, we discuss lncRNAs which are involved in neuroprotection mechanisms and up-to-date knowledge regarding lncRNAs as promising therapeutic approaches in IS **(**Fig. 2**)**.

### Article Search Process

**Fig. 1:**
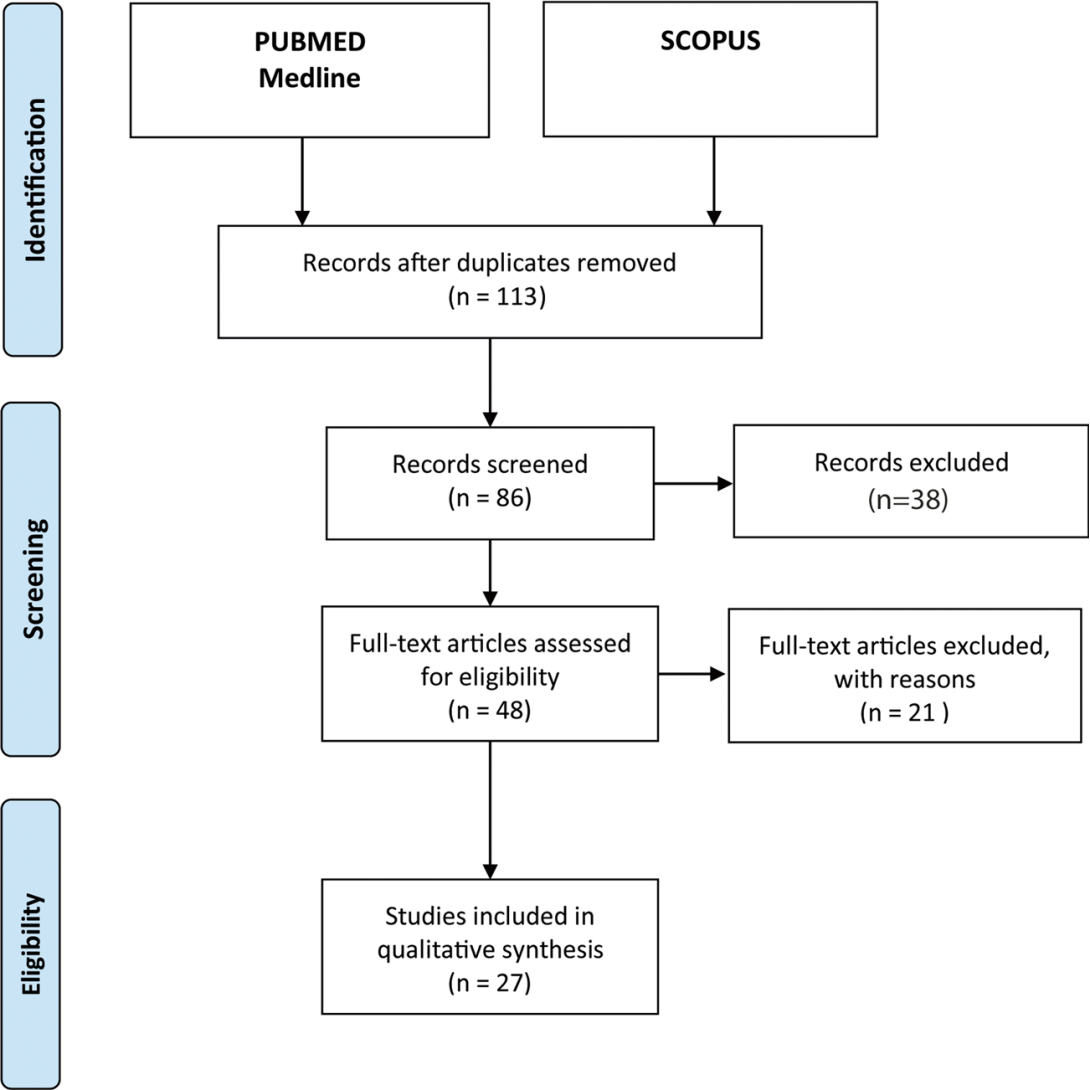
Article selection flowchart. The figure schematically depicts the article selection process, from literature search, through the screening, up to the final assessment of eligibility

Electronic databases Pubmed and Scopus were searched between May 2020 and June 2020 and original studies were reviewed to evaluate the role of circulating lncRNAs in IS. Review articles and meta-analysis were incorporated in this as well as their secondary references for possible inclusion. Titles and abstracts were screened by two independent operators. The following search syntax was used: “Search (“long non coding RNAs” [MeSH Terms] or “lncRNA” [MeSH Terms] or “long non-coding RNA” [MeSH Terms] or “circulating lncRNA” [MeSH Terms] or “circulating long non coding RNA” [MeSH Terms]) and (“treatment” [MeSH Terms] or “therapeutic” [All Fields]) and (“ischemic stroke” [MeSH Terms] or “ischemia” [MeSH Terms] or “stroke” [MeSH Terms] or “ischaemic stroke” [MeSH Terms] or “ischaemia” [All Fields])” (Fig. 1).

**Fig. 2:**
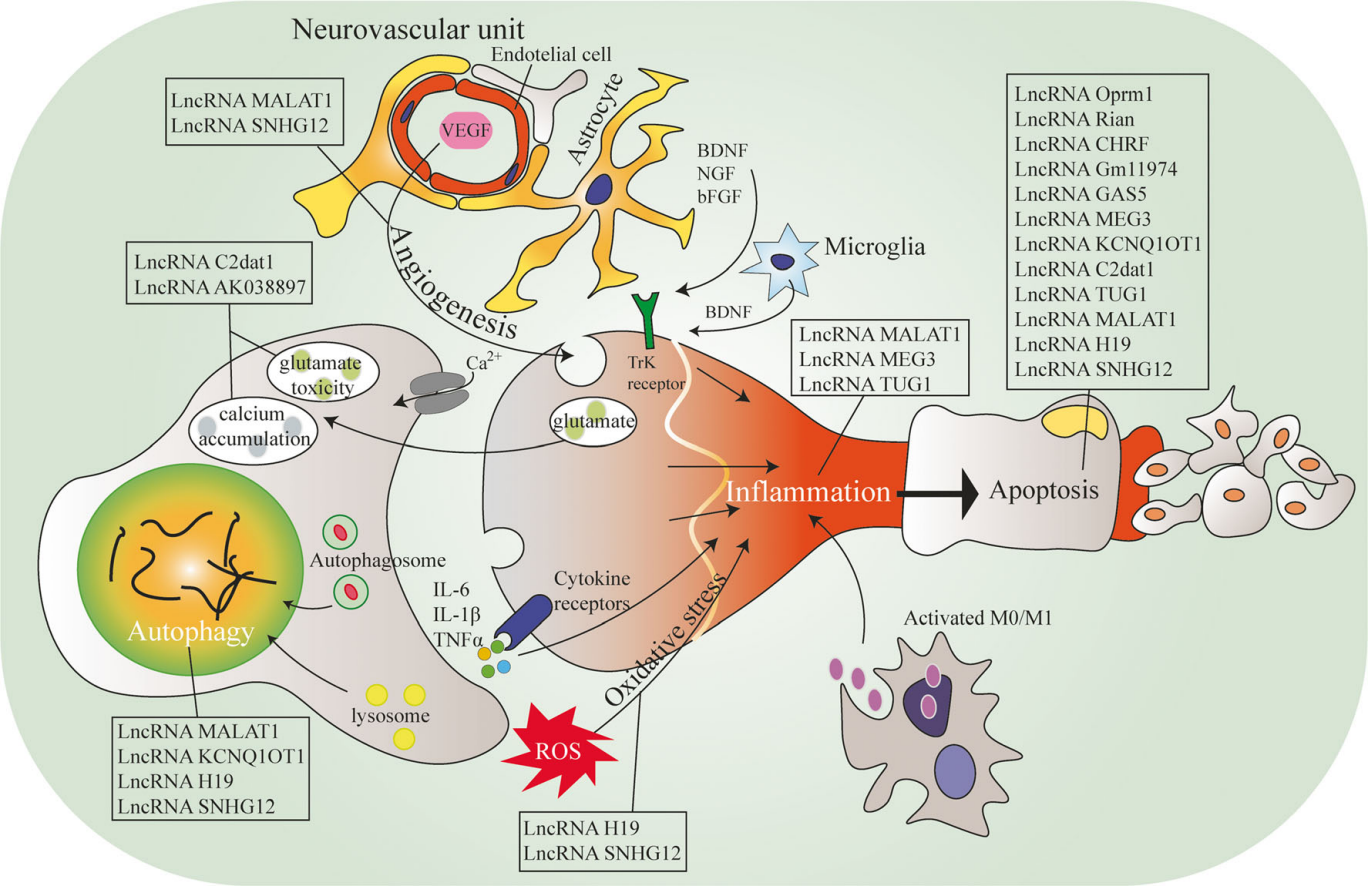
The role of lncRNAs in the pathophysiology of neurodegeneration. Abbreviations: BDNF, brain-derived neurotrophic factor; bFGF, basic fibroblast growth factor; C2dat1, CAMK2D-associated transcript; CHRF, cardiac hypertrophy–related factor; GAS5, growth arrest-specific transcript 5; IL-1β, interleukin-1β; IL-6, interleukin-6; KCNQ1OT1, potassium voltage-gated channel subfamily Q member1 opposite stand 1; MALAT1, metastasis-associated lung adenocarcinoma transcript 1; MEG3, maternally expressed gene 3; M0/M1, microglia non-activated/pro-inflammatory; NGF, nerve growth factor; Rian, RNA imprinted and accumulated in nucleus; SNHG12, small nucleolar RNA host gene 12; TNFα, TNF receptor-associated factor; TrK, tropomyosin receptor kinase; TUG1, taurine-upregulated gene 1; VEGF, vascular endothelial growth factor

**Fig. 3:**
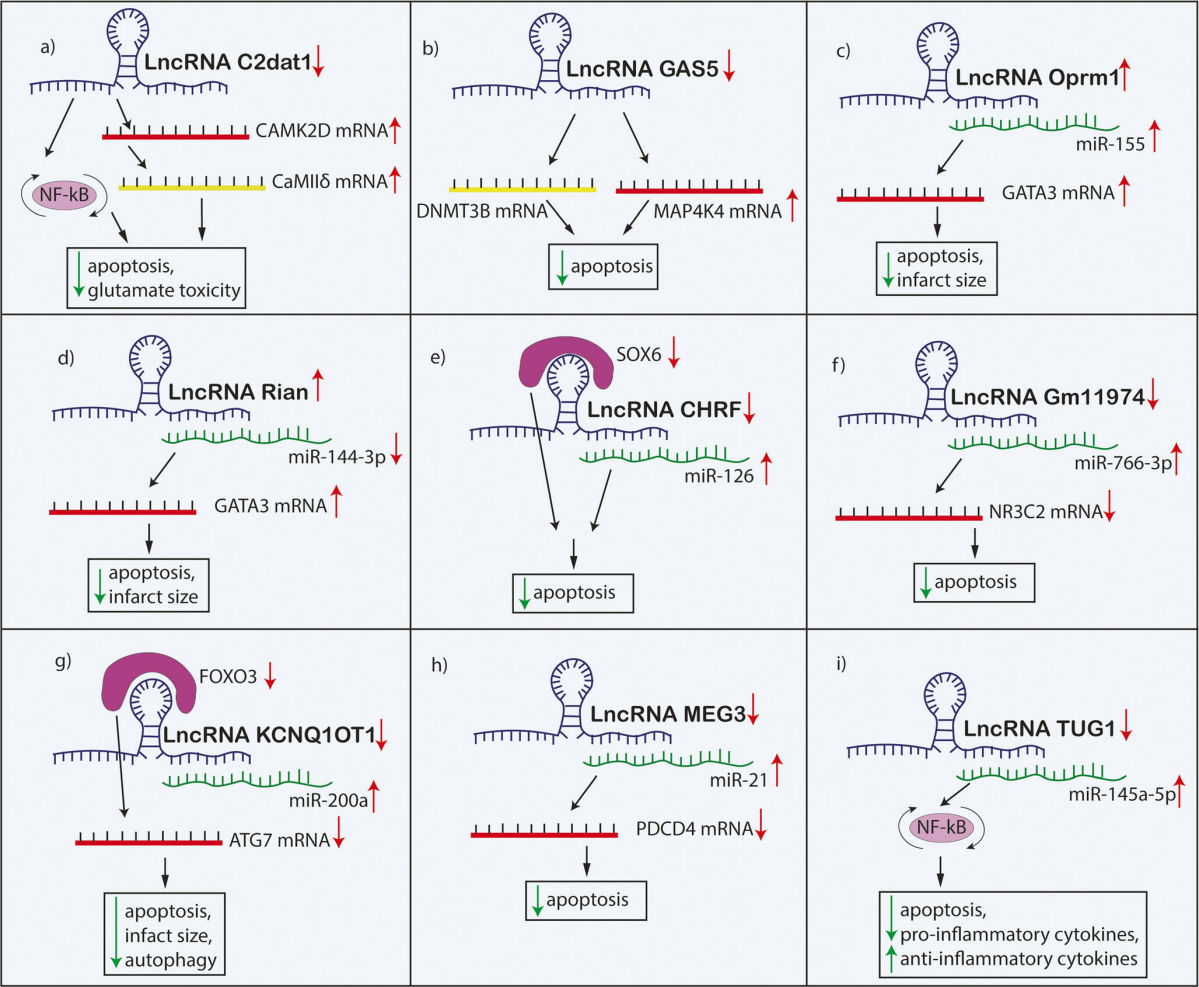
The mechanisms by which lncRNAs contribute to the neuronal cell death process in ischemic stroke. Abbreviations: Oprm1, μ-opioid receptor 1; GATA3, GATA binding protein 3; Rian, RNA imprinted and accumulated in nucleus; CHRF; cardiac hypertrophy–related factor; SOX6, sex-determining region Y-box 6; NR3C2, nuclear receptor subfamily 3 group C member 2; GAS5, growth arrest-specific transcript 5; DNMT3B, DNA methyltransferase 3B; MAP4K4, mitogen-activated protein kinase kinase kinase kinase 4; MEG3, maternally expressed gene 3; PDCD4, programmed cell death 4; KCNQ1OT1, potassium voltage-gated channel subfamily Q member1 opposite stand 1; FOXO3, forkhead box O3; ATG7, autophagy-related 7; C2dat1, CAMK2D-associated transcript; CAMK2D, calcium/calmodulin-dependent protein kinase II; CaMIIδ, calcium/calmodulin-dependent protein kinase IIδ; NF-κB, nuclear factor kappa B; TUG1, taurine-upregulated gene 1

**Fig. 4:**
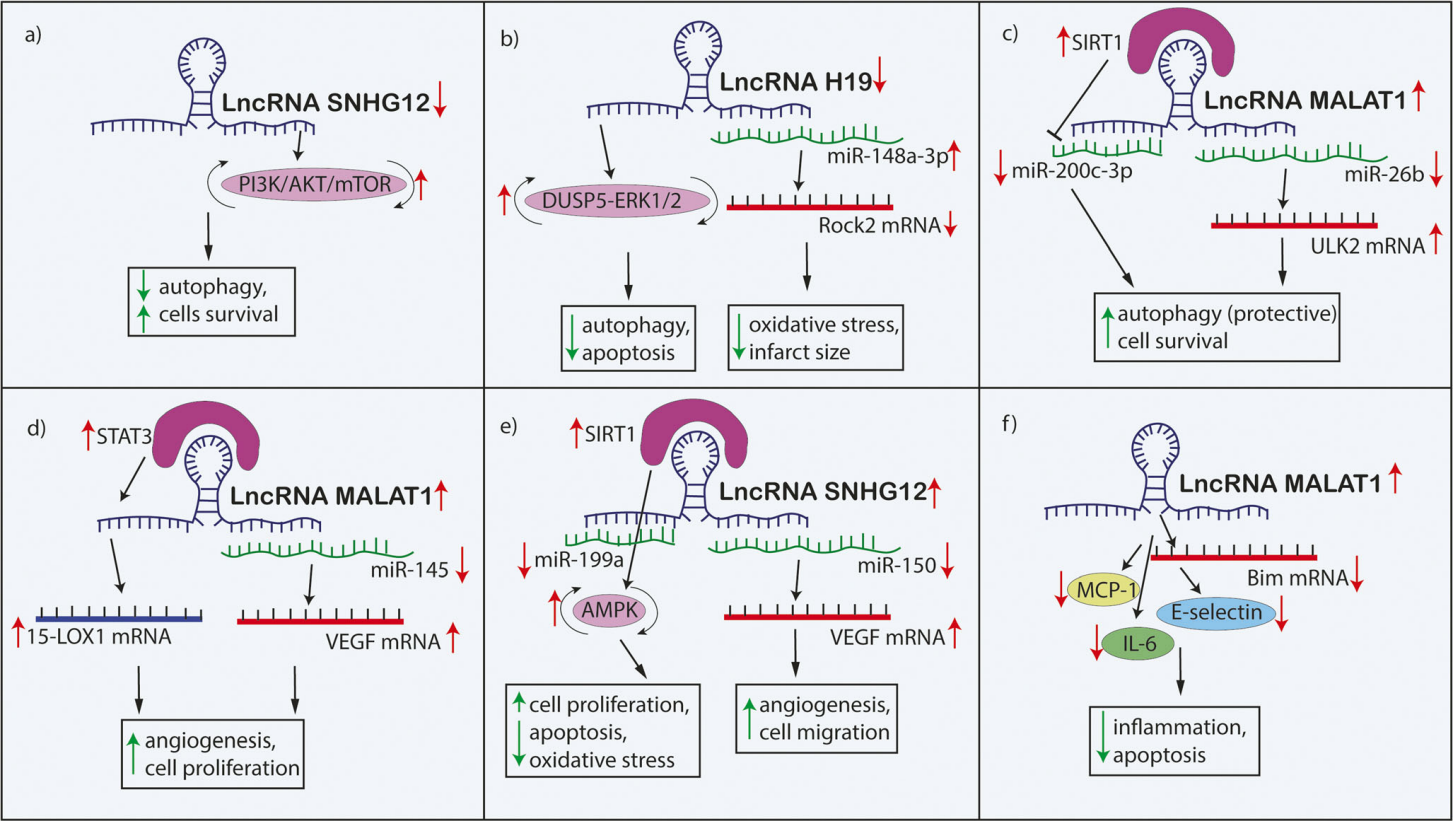
The mechanism of lncRNAs MALAT1, H19, and SNHG12 in ischemic stroke pathogenesis. Abbreviations: MALAT1, metastasis-associated lung adenocarcinoma transcript 1; ULK2, Unc-51-like autophagy activating kinase 2; 15-LOX1, 15-lipoxygenase 1; VEGF, vascular endothelial growth factor; MCP-1, monocyte chemoattractant protein-1; IL-6, interleukin-6; DUSP5-ERK1/2, dual-specificity phosphatase 5-extracellular signal-regulated kinase ½; Rock2, Rho-associated protein kinase 2; SIRT1, sirtuin 1; SNHG12, small nucleolar RNA host gene 12; AMPK, AMP-activated protein kinase; PI3K, phosphoinositide 3-kinase; AKT, protein kinase B; mTOR, the mammalian target of rapamycin

**Fig. 5:**
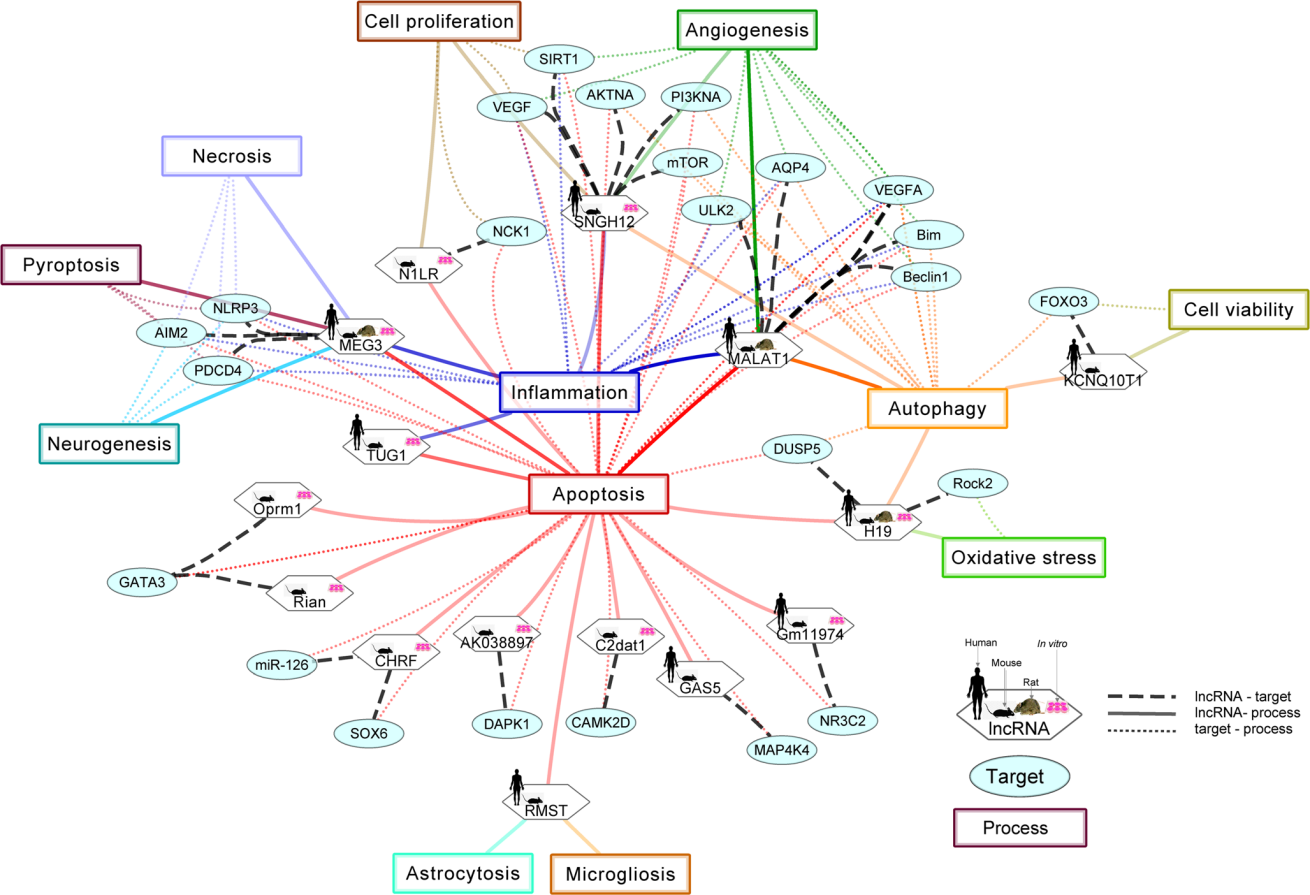
Summary network graph showing information gained from studies investigating lncRNAs as potential novel therapeutic approaches in ischemic stroke

## LncRNAs Regulating Glutamate Excitotoxicity in I/R Injury as a Key Factor in IS Severity and Neurological Impairment

The precise underlying mechanisms involved in the pathophysiology of cerebral I/R injury remain relatively undiscovered. Nevertheless, lncRNAs which are able to modulate reperfusion injury, associated with enhanced neuronal cell death and hemorrhagic transformation, seem to play a significant role in IS treatment [12]. Studies showed that ischemic injury leads to excessive glutamate release which activates N-methyl-D-aspartate (NMDA) receptors and causes an excessive calcium influx in neurons. Overstimulation of NMDA receptors by glutamate excitotoxicity is one of the trigger factors in initiating neuronal cell apoptosis [13]**.** Moreover, cerebral blood reperfusion of ischemic areas leads to enhanced calcium overload and further brain tissue damage. Excessive calcium accumulation results in the activation of calcium/calmodulin-dependent protein kinase II (CaMKII), a family of multifunctional serine/threonine kinases involved in IS pathogenesis [14].

### LncRNA C2dat1

Previous studies showed that CaMKII can be regulated by lncRNAs. One of the reports showed that the lncRNA *CAMK2D*-associated transcript (C2dat1) is able to regulate CaMKIIδ, a CaMKII isoform, by targeting *CAMK2D* [14]. The levels of lncRNA C2dat1 and *CAMK2D* were upregulated both in vivo and in vitro models of IS. Moreover, CaMKIIδ was upregulated in the peri-infarct region, but was downregulated in the ischemic core. C2dat1 inhibition caused increased neuronal death and decreased levels of both *CAMK2D* and CaMIIδ. Prolonged inhibition of CaMII promoted neuronal apoptosis by increasing the glutamate toxicity vulnerability, whereas short-term inhibition protected against glutamate toxicity. Importantly, C2dat1 promoted neuronal survival via activation of nuclear factor kappa B (NF-κB) signaling cascade, which may suggest C2dat1 is a promising therapeutic approach for ischemia [14]. On the other hand, another report showed that the inhibition of CaMKII can prevent 30–70% of ischemia-induced neuronal death [15]**.** These discrepancies are probably caused by the fact that chronic inactivation of CaMKII increases the susceptibility of neurons to glutamate toxicity, whereas acute inactivation protects against glutamate, hypoxia, and hypoglycemia [16, 17]. Moreover, upregulation of CaMKIIδ in the peri-infarct region and its downregulation in ischemic core could be explained by the fact that *CAMK2D* is inactivated in a time- and location-dependent manner and the neurological deficit correlates with the rate of *CAMK2D* inactivation [14]. Consequently, early treatment with lncRNA C2dat1 can reduce the severity of neurological impairment after IS and should be further investigated in preclinical research (Fig. 3a).

### LncRNA AK038897

Another lncRNA that may influence calcium homeostasis in IS is AK038897. AK038897 acts like a sponge by binding to miR-26a-5p and thus interfering with its target interactions. *DAPK1*, a serine/threonine kinase, was determined as a target gene of miR-26a-5p. *DAPK1* can be regulated by calcium/calmodulin and is associated with excessive calcium influx through glutamate release, leading to mitochondrial dysfunction and neuronal membrane folding. AK038897 silencing could diminish *DAPK1* levels via miR-26a-5p upregulation, contributing to protection against I/R injury. This observation was confirmed in vivo, as AK038897 knockdown resulted in reduced infarcted area and neurological impairment [18, 19]**.** As previous studies reported that *DAPK1* deletion prevents calcium overload and protects against ischemic neuronal death in IS [20], downregulation of *DAPK1* by AK038897 can potentially protect against I/R injury. Additionally, the transfection of *DAPK1* caused more severe cerebral tissue damage in the IS mice model compared to the sham group [13, 18]. Thus, these findings indicate that lncRNAs are able to regulate *DAPK1* and can be promising therapeutic approaches in IS.

Collectively, mentioned studies emphasized the role of mitochondrial dysfunction, excessive glutamate, and calcium accumulation in neuronal injury, indicating that lncRNAs which are capable of protecting mitochondrial membranes and controlling glutamate receptors and calcium channels can be promising therapeutic approaches in IS **(**Table 1**).**

**Table 1:** Studies evaluating lncRNAs as a potential novel therapeutics in ischemic stroke

Ref.	LncRNA	Down- or upregulation in IS	Regulated genes	Pathophysiological mechanism (inflammation/atrophy)	Species/animal model (tissue, cell line)/human study (serum/CSF/tissue)	Axis	Conclusion
[21]	Gm11974	Upregulated	NR3C2	Apoptosis	HEK293T and N2a cells	lncRNA Gm11974/miR-766-3p/NR3C2	Silencing of lncRNA Gm11974 may have therapeutic effect in IS
[3]	MALAT1	Upregulated	AQP4	Apoptosis	C57BL/6 J mice, primary astrocytes	lncRNA MALAT1/miR-145/AQP4	Silencing of lncRNA MALAT1 protects against I/R injury
[22]	SNHG12	Upregulated	/	Autophagy	Mice, SHSY5y cells	/	LncRNA SNHG12 can alleviate I/R injury via autophagy induction
[23]	H19	Upregulated	Rock2	Oxidative stress	C57BL/6 mice, N2a cells	lncRNA-H19/miR-148a-3p/Rock2	LncRNA H19 is involved in metformin neuroprotective effect on I/R injury
[24]	MALAT1	Upregulated	Bim	Apoptosis, inflammation	C57BL/6 J mice, mouse primary BMECs, N2a cells	/	MALAT1 regulates apoptosis and inflammation in IS
[25]	SNHG12	Upregulated	SIRT1	Cell proliferation, apoptosis	Mouse primary hippocampal neuronal cells, N2a cells	lncRNA SNHG12/miR199a/SIRT1/AMPK	SNHG12 alleviates I/R injury by regulation of neuronal cell apoptosis and proliferation
[26]	SNHG12	Upregulated	SNHG12	Apoptosis, inflammation	Primary neurons of C57 mice	Akt signaling pathway	SNHG12 is able to reduce IS injury through apoptosis, inflammation reduction
[2]	MEG3	Upregulated	/	Apoptosis, necrosis, neurogenesis	Male Sprague-Dawley rats	Wnt/β-catenin signaling pathway	Silencing of MEG3 is able to involve neurogenesis and reduce neurological impairment
[27]	RMST	Upregulated	/	Apoptosis, microgliosis, astrocytosis	male C57/BL6 mice, mice primary hippocampal neurons, and human plasma		RMST silencing reduce infarct size and neurological impairment
[28]	MEG3	Upregulated	AIM2	Pyroptosis, inflammation	Male Sprague-Dawley rats, human neuroblastoma cell lines SK-N-SH and SH-SY5Y	lncRNA MEG3/miR-485/AIM2	Silencing of MEG3 can decrease pyroptosis and inflammation
[29]	H19	Upregulated	DUSP5	Autophagy, apoptosis	SH-SY5Y cells, rats	lncRNA H19/DUSP5-ERK1/2	H19 induces I/R injury via autophagy activation. H19 gene variation increases risk of IS in human patients
[6]	Oprm1	Downregulated	GATA3	Apoptosis	N2a cells, C57/B6 mice	lncRNA Oprm1/miR-155/GATA3	Overexpression of lncRNA Oprm1alleviates I/R injury via apoptosis reduction
[30]	CHRF	Upregulated	SOX6	Apoptosis	Male C57BL/6 J mice, N2a cells	lncRNA CHRF/miR-126/SOX6	Silencing of lncRNA CHRF alleviates ischemic neuronal death and neurological impairment
[31]	KCNQ10T1	Upregulated	ATG7	Autophagy	Plasma from patients after IS, Male C57BL/6 J mice, N2a cells	KCNQ10T1/miR-200a/FOXO3/ATG7	Silencing of KCNQ10T1 alleviates I/R injury via autophagy inhibition
[19]	N1LR	Upregulated = in mild ischemia; down regulated = in severe ischemia	Nck1 (but not enough evidence to prove this)	Cell proliferation apoptosis	N2a cells, male Sprague-Dawley rats, male C57BL/6 J mice		N1LR promotes neuroprotection via the inhibition of p53 phosphorylation serine 15
[32]	MALAT1	Upregulation	MALAT1	Angiogenesis	Male C57BL/6 J mice, BMECs	lncRNA MALAT1/15-LOX1/STAT3	MALAT1 promotes angiogenesis following OGD/R
[13]	C2dat1	Upregulated	CAMK2D	Apoptosis	Male C57BL/6 J mice, N2a cells	NF-kB signaling pathway	C2dat1promote neuronal survival by CAMK2D and NF-kB signaling pathway
[18]	GAS5	Upregulated	MAP4K4	Apoptosis	C57BL/6 mice, serum of patients after IS		Silencing of GAS5 reduces neuronal apoptosis and neurological impairment via DNMT3B-mediated MAP4K4 methylation
[33]	TUG1	Upregulated	TUG1	Apoptosis inflammation	SH-SY5Y human neuroblastoma cells, BV-2 microglial cells	lncRNA TUG1/mir-145a-5p and NF-kB signaling pathway	lncRNA TUG1 can regulate microglial polarization and inhibit inflammatory cytokines in IS
[34]	MEG3	Upregulated	PDCD4	Apoptosis	Male C57BL/6 J mice, N2a cells	lncRNA MEG3/miR-21/PDCD4	Silencing of MEG3 protects against ischemic injury and improves neurological functioning
[16]	AK038897	Upregulated	DAPK1	Apoptosis	Male C57BL/6 J mice, N2a cells	lncRNA AK038897/miR-26a-5p/DAPK1	Silencing of AK038897 protects against brain injury and neurological deficits
[35]	Rian	Downregulated	GATA3	Apoptosis	C57BL/6 mice, N2a cells	lncRNA Rian/miR-144-3p/GATA3	Rian overexpression reduce cell apoptosis and infarct size and improve neurological functioning
[36]	SNHG12	Upregulated	VEGF	Angiogenesis	Male C57BL/6 mice, BMECs	lncRNA SNHG12/miR-150/VEGF	SNHG12 overexpression increased vascular density in the infarct border zone and improved neurological function
[37]	MALAT1	Upregulated	VEGFA	Apoptosis, angiogenesis	HBMECs, serum from patients after IS, rats	lncRNA MALAT1/miR-205-5p/VEGFA	MALAT1 can protect angiogenesis function under OGD/R conditions in vitro
[4]	MALAT1	Upregulated	ULK2	Autophagy	C57BL/6 J mice, BMECs	lncRNA Malat1/miR-26b/ULK2	Malat1 is an autophagy inducer protects against OGD/R-induced injury
[38]	MALAT1	Upregulated	Beclin1	Autophagy	Male C57BL/6 J mice, mice primary cortical neurons	lncRNA Malat1/miR-30a/Beclin1	Silencing of MALAT1 may protect neurons via suppression of Beclin1-dependent autophagy
[39]	SNHG12	Upregulated	/	Apoptosis, autophagy	BMECs, MSCs	PI3K/AKT/mTOR	Silencing of SNHG12 accelerated the effect of MSCs on reducing autophagy and apoptosis

*LncRNA* long non-coding RNA, *N2a* neuro-2A neuroblastoma cells, *AQP4* aquaporin 4, *MALAT1* metastasis-associated lung adenocarcinoma transcript 1, *SNHG12* small nucleolar RNA host gene 12, *Rock2* Rho-associated protein kinase 2, *MiRNA* microRNA, *SIRT1* sirtuin 1, *IS* ischemic stroke, *I/R* ischemia/reperfusion, *MEG3* maternally expressed gen 3, *BBB* blood-brain barrier, *RMST* rhabdomyosarcoma 2-associated transcript, *AIM2* absent in melanoma 2, *GATA3* GATA binding protein 3, *CHRF* cardiac hypertrophy–related factor, *SOX6* sex-determining region Y-box 6, *AIS* acute ischemic stroke, *KCNQ1OT1* potassium voltage-gated channel subfamily Q member1 opposite stand 1, *FOXO3* forkhead box O3, *OGD/R* oxygen and glucose deprivation/reperfusion, *15-LOX1* 15-lipoxygenase, *VEGF* vascular endothelial growth factor, *STAT3* signal transducer and activator of transcription 3, *C2dat1* CAMK2D-associated transcript, *CAMK2d* calcium/calmodulin-dependent kinase II, *NF-kB* nuclear factor kappa B, *DNMt3B* DNA methyltransferase 3B, *GAS5* growth arrest-specific transcript 5, *MAP4K4* mitogen-activated protein kinase kinase 4, *TUG1* taurine upregulated gene 1, *PDCD4* programmed cell death 4, *DAPK1* death-associated protein kinase 1, *Rian* RNA imprinted and accumulated in nucleus, *BMECs* brain microvascular endothelial cells, *MSCs* mesenchymal stem cells, *VEGF* vascular endothelial growth factor

## LncRNAs Involved in Signaling Pathways Responsible for Neuronal Cell Death

Rising evidence confirms the crucial role of lncRNAs which acts as a sponge by binding to miRNAs and thus they are able to target multiple neural pathways. Hence, the main objective is to identify and understand those axes and find a proper way to alter its pathological effects in ischemic tissue.

### LncRNA GAS5

The role of lncRNA GAS5 and its relationship with *MAP4K4* gene was investigated by several experimental strategies. Overexpression of *MAP4K4* reduced the apoptotic rate of oxygen-glucose deprivation/reperfusion (OGD/R, an in vitro model of IS)-induced neurons. In silico analysis predicted that lncRNA GAS5 interacts with *MAP4K4*. Moreover, *DNMT3B* was identified as a direct target gene of GAS5. LncRNA GAS5 administration downregulated *MAP4K4* expression through DNMT3B recruitment in mice primary cortical neurons. Furthermore, overexpression of lncRNA GAS5 increased the neuronal apoptosis rate. Additionally, lncRNA GAS5 administration resulted in a larger area of cerebral infarction and exacerbated neurological test results in animal models. Consequently, this study indicated that the inhibition of GAS5 in IS could potentially reduce neuronal apoptosis, reduce infarct size and improve neurological functioning via the MAP4K4/DNMT3B axis [40] **(**Fig. 3b**).**

### LncRNA N1LR

Genome-wide lncRNA expression analysis revealed that lncRNA N1LR is significantly altered during I/R injury. The expression of N1LR was increased after mild ischemic injury, but reduced after more severe ischemic injury. Additionally, N1LR was mainly located in the cytoplasm in undamaged tissue, however after I/R injury N1LR was mostly accumulated in the nucleus. Overexpression of lncRNA N1LR accelerated cell cycle progression, promoted proliferation, and inhibited apoptosis after OGD/R injury (in vitro model). Moreover, overexpression of N1LR resulted in reduced infarct volume and neurological deficits, while silencing caused the opposite effect in the animal model. Furthermore, N1LR administration significantly reduced neuronal apoptosis. Importantly, N1LR inhibited phosphorylation of the p53 protein, suggesting that the protective effect of lncRNA N1LR against I/R injury may be due to the blockage of p53 phosphorylation [35].

Overall, more and more lncRNAs are being characterized functionally in a different neuronal cell context. Previous studies have shown that lncRNAs, as a part of complex signaling pathways, are able to regulate neuronal apoptosis, inflammation, and IS severity. Notably, the complexity of those axes allows for their modification at various stages. Further studies are needed on the cellular effects that might be caused by targeting lncRNAs as a part of the multifunctional signaling pathways involved in IS (Table 1).

### LncRNA Oprm1

The role of lncRNA Oprm1 in cerebral I/R injury was studied both in vitro and in vivo models of IS. Following the reperfusion time, the lncRNA Oprm1 expression was decreased in vitro. The overexpression of Oprm1 decreased the apoptosis rate as well as reduced the infarct size and improved the neurological functioning of I/R model experimental animals. As Oprm1 can directly target miR-155, whereas miR-155 can directly target G*ATA3*, Oprm1 demonstrated a protective role by sponging miR-155, thus regulating *GATA3* expression. *GATA3* is involved in neuronal differentiation and survival of sympathetic progenitors and neurons in embryonic chromaffin cells in in vitro analysis [41]. Collectively, this study suggests the importance of the Oprm1/miR-155/*GATA3* axis in neuronal cell death [6]. Importantly, not only Oprm1 treatment but also miR-155 alone treatment decreased the infarct size; moreover it also reduced the volume of hemorrhagic transformation of IS, suggesting that Oprm1/miR-155 axis can exert more potent protective effect than miR-155 alone treatment [30] (Fig. 3c).

### LncRNA Rian

Similarly, the role of lncRNA Rian and its relation with miR-144-3p levels and *GATA3* were studied in IS experimental models. Rian, as well as *GATA3*, were downregulated in both in vitro and in vivo models, whereas miR-144-3p was upregulated. Overexpression of Rian reversed miR-144-3p-induced cerebral ischemic injury in mice. Moreover, overexpression of lncRNA Rian reduced the neuronal apoptosis and infarct size and improved the neurological score. *GATA3* was identified as a target gene of miR-144-3p and was involved in the miR-144-3p-mediated injury mechanism. Importantly, lncRNA Rian also regulates *GATA3* and overturns the miR-144-3p-induced suppression of *GATA3*. Collectively, lncRNA Rian can potentially suppress miR-144-3p-mediated neuronal loss via Rian/miR-144-3p/*GATA3* axis. Therefore, it can be treated as an important therapeutic approach and should be further investigated in preclinical research [21] (Fig. 3d).

### LncRNA CHRF

LncRNA CHRF was found to play an important role in the processes of cardiac dysfunction and in the regulation of myocardiocyte death after reperfusion [42]. Besides, the importance of CHRF on I/R injury in IS was also determined. LncRNA CHRF was found to be upregulated after I/R injury and could directly bind to miR-126, whereas miR-126 was downregulated in I/R injury. Moreover, CHRF can directly target *SOX6* which is associated with diverse apoptosis mechanisms and thus can modulate ischemic cell apoptosis. Knockdown of CHRF significantly increased miR-126 and decreased *SOX6* expression. Thus, inhibition of CHRF can reduce neuronal damage and neurological impairment by targeting the miR-126/*SOX6* cascade [43]. It is important to note that miR-126 was intensively studied in the context of platelet function in patients with hyper platelet activity and growing evidence showed that miR-126 can be a promising biomarker in platelet activation [8, 9, 44]. Platelet activation and aggregation are critical in the pathogenesis of IS. Patients with IS exhibit hyper-reactive platelets compared to healthy individuals [45]. Therefore, future analysis should aim to analyze the importance of CHRF/miR-126 sponging regarding platelet activation in patients with IS (Fig. 3e).

### LncRNA Gm11974

The function of another lncRNA Gm11974 was evaluated in the context of neuronal cell death in an IS in vitro model. Knockdown of Gm11974 caused a significant decrease in cell death rate and apoptosis as well as increased cell viability and protected mitochondrial membrane potential. Furthermore, the inhibition of Gm11974 increased the expression of miR-766-3p in vitro, demonstrating that Gm11974 could potentially negatively regulate miR-766-3p. Moreover, *NR3C2* was negatively regulated by miR-766-3p*.* Knockdown of lncRNA Gm11974 decreased cell apoptosis through miR-766-3p upregulation, which antagonized *NR3C2*, demonstrating the role of Gm11974/miR-766-3p/*NR3C2* axis in I/R injury. Consequently, these results indicate that silencing of Gm11974 may protect against cerebral reperfusion injury; thus this lncRNA should be further investigated for clinical application as a therapeutic approach in IS [46] (Fig. 3f).

### LncRNA MALAT1

LncRNA MALAT1 and its association with *AQP4* in cerebral I/R injury was determined in OGD/R astrocyte cell and middle cerebral artery occlusion (MCAO) mouse models. MALAT1 expression was upregulated in both models. Knockdown of MALAT1 increased the survival of astrocytes and decreased the apoptotic rate. Additionally, knockdown of *AQP4*, a transmembrane protein associated with brain edema and apoptosis, caused a decrease in cytotoxicity and reduced apoptosis of astrocytes. Moreover, cell damage was alleviated after the *AQP4* knockdown. As it was determined that MALAT1 can act as ceRNA for miR-145, MALAT1 positively regulated *AQP4* via miR-145 downregulation. Altogether, the study validated that MALAT1 silencing protects against cerebral I/R injury by regulating the miR-145/AQP4 axis [3].

To sum up, lncRNA Oprm1 and lncRNA Rian demonstrate protective properties against I/R injury through *GATA3* modulation. Besides, lncRNA CHRF, MALAT1, and Gm11974 also present positive effects on pathological processes in I/R injury. Consequently, all the mentioned lncRNAs show great potential as novel therapeutic approaches in IS and therefore merit further investigation in preclinical research (Table 1).

## Ischemia/Reperfusion Injury Exacerbating Factors as Therapeutic Approaches

Following cerebrovascular thrombosis, reperfusion of tissues by the restoration of adequate oxygen supply may cause further damage to brain tissue and should be distinct from the injury that is caused by ischemia. Changes that occur in brain tissue under deprivation of oxygen and other nutrients cause oxidative and inflammatory damage of neuronal cells [47]. This oxidative stress is likely to contribute to autophagy activation, which determines subsequent processes in the I/R injury region [48]. The significance of autophagy in IS will be broadly described in this section, as the final effect on neurons is highly dependent on the phase of reperfusion. Previous evidence suggests that autophagy, angiogenesis, as well as oxidative stress have a critical effect on I/R injury and can be moderated by lncRNAs [49]. Exploring lncRNAs in the abovementioned processes may help to understand the pathomechanisms involved in IS and thus recovery networks induced after ischemia.

### Autophagy

Autophagy, as a lysosomal degradation pathway, is responsible for the recycling of damaged and aged cellular components and is crucial for neuronal hemostasis [50]. As described by Wang et al., the activation of the autophagy pathway in the brain upon ischemic stimuli can be a double-edged sword for neural survival after IS. To date, some studies have demonstrated a protective role of autophagy in IS while others have shown damaging effects [51]. Importantly, several authors have underlined that autophagy can be neuroprotective during early stages of ischemia; however, prolonged ischemia leads to neurotoxic autophagy [52]. Besides, it was also hypothesized that autophagy can show protective effects during ischemia, whereas it can be detrimental during reperfusion period [22]. Autophagy activity might be measured using autophagy-related gene expression assessment such as *Beclin-1*, *LC3 I*, and *LC3 II* [39]. The role of lncRNAs in the regulation of autophagy has been extensively investigated in previous years; however, the majority of research concentrated on myocardial infarction and its reperfusion injury [29, 31, 53]. The association of lncRNAs and autophagy-related genes in IS remains unclear and should be further investigated.

#### LncRNA SNHG12

The expression of lncRNA SNHG12 was shown to be upregulated in cerebral I/R injury and associated with enhanced autophagy activation and increased neuronal cell survival both in in vitro and in vivo analysis [22, 54]. Additionally, the role of SNHG12 as a regulator of mesenchymal stem cell (MSC) function in I/R injury was also determined. Downregulation of SNHG12 was found to potentiate the ability of MSCs to reduce autophagy via PI3K/AKT/mTOR axis. In I/R injury treated with SNHG12-modified MSCs, autophagy, apoptosis, and infarcted regions were reduced [55]**.** Collectively, lncRNA SNHG12 might be an important regulator of autophagy in IS and possesses therapeutic potential in I/R injury. Nevertheless, as previous studies demonstrated, the neuroprotective role of lncRNA SNHG12 in IS can be achieved by both reduction and enhancement of autophagy (Fig. 4a).

#### LncRNA H19

LncRNA H19 was found to play an important role in stimulating autophagy in cerebral I/R injury, as significant overexpression of lncRNA H19 and autophagy activation in cerebral tissue were observed in previous experiments. Further analysis showed that lncRNA H19 decreases cell viability via autophagy promotion through inhibition of the DUSP5-ERK1/2 pathway. LncRNA H19 inhibition resulted in reduced autophagy and neuronal cell apoptosis in in vitro analysis. Furthermore, the study revealed that *H19* gene variations are associated with increased risk of IS. Consequently, lncRNA H19 seems to play an important role in I/R injury via autophagy activation and its inhibition may have therapeutic effect on cerebral tissue in IS [56] (Fig. 4b).

#### LncRNA KCNQ1OT1

Another lncRNA KCNQ1OT1 was upregulated in IS patients’ plasma and was correlated with IS severity. Administration of KCNQ1OT1 inhibitor in mice decreased *ATG7* (autophagy-related gene) expression, which resulted in reduced infarct volume, neurological impairment, and inhibited autophagy. Similar results associated with autophagy were observed in vitro, but importantly, activated autophagy promoted neuronal apoptosis and could be inhibited by KCNQ1OT1 knockdown, suggesting its protective role. MiR-200a was detected as a direct target of KCNQ1OT1. MiR-200a can regulate FOXO3 expression, which is able to regulate *ATG7*, and thus autophagy. Knockdown of KCNQ1OT1 increased miR-200a and decreased FOXO3 expression. Collectively, inhibition of KCNQ1OT1 is associated with reduced autophagy and therefore neuronal dysfunction through the miR-200a/FOXO3/*ATG7* axis [57] (Fig. 3g).

As above mentioned, the role of autophagy in IS is unclear. There are more than 300 studies considering the role of autophagy in IS, some demonstrating autophagy inhibitors to induce a neuroprotective effect and reduce infarct size and apoptosis rate [38, 51, 58], while others reported autophagy inhibition to result in increased neuronal cell apoptosis and neurological deficits [38, 51, 58, 59]. Based on these reports, it can be hypothesized that lncRNAs enhance autophagy during early stages of ischemia, while reducing autophagy in prolonged ischemia and I/R injury. Therefore lncRNAs should be a target of future research investigating autophagy effects on ischemia.

#### LncRNA MALAT1

The role of lncRNA MALAT1 is broadly emphasized in various processes following IS, including autophagy regulation in the I/R injured brain area. To date, there are three studies that have investigated the relationship of MALAT1 and autophagy in IS. Overexpression of MALAT1 was able to enhance autophagy and improve the neuronal survival in an in vitro model of IS. The study revealed that MALAT1 acted as an endogenous sponge of miR-26b and downregulated its expression. Prediction analysis showed that *ULK2*, autophagy-related gene, is a direct target gene of miR-26b. Overexpression of MALAT1 upregulated *ULK2* via downregulation of miR-26b and increased autophagy rate and neuronal survival. Overall, the results suggest that MALAT1/miR-26b/*ULK2* axis promotes autophagy and survival [4]. Similarly, another study showed that MALAT1 was upregulated in the IS in vitro model and its overexpression was associated with enhanced autophagy and increased cell survival. Furthermore, MALAT1 reduced the expression of miR-200c-3p. MALAT1 directly binds to miR-200c-3p and cells that were treated with MALAT1 presented reduced apoptosis. Importantly, MALAT1 induces SIRT1 (an autophagy activator and neuroprotector factor) by blocking miR-200c-3p expression [32, 37] (Fig. 4c). Contrary to previous studies, Guo et al. reported that downregulation of MALAT1 reduced autophagy and thus promoted neuronal cell survival after IS both in in vitro and in vivo models. The study showed that silencing of MALAT1 upregulated miR-30a. *Beclin-1*, an autophagy biomarker, was a target gene of miR-30 and was negatively regulated by miR-30. Thus, silencing of MALAT1 suppressed *Beclin-1*-dependent autophagy via *Beclin-1* downregulation [36]. As mentioned previously, the contrary results can be explained by the unclear role of autophagy in IS, as it might have either positive or negative effects on neuronal cells. Collectively, lncRNA MALAT1 regulation of autophagy in I/R injury can promote both neuroprotection and neuronal death. Thus, further studies are needed in this topic to clarify the underlying mechanisms.

### Angiogenesis

Neuronal angiogenesis is a multi-step process, involving the proliferation of human brain microvascular endothelial cells (HBMECs) which differentiate into tubular micro-vessels. Several reports have demonstrated the promotion of angiogenesis in specific brain regions after ischemic injury. Furthermore, there is a correlation between angiogenesis and improved neurological functioning after IS [60]. Even though the role of lncRNAs in angiogenesis after I/R injury remains unclear, there are few studies emphasizing the importance of MALAT1 and SNHG12 in the process of angiogenesis in IS.

#### LncRNA MALAT1

It was previously found that MALAT1 is upregulated in both in vitro and animal models as well as in patients with IS. MALAT1 downregulation was related to reduced EC proliferation, cell migration, and reduced CD31 expression (an angiogenesis-associated marker) leading to reduced angiogenesis capacity in vitro. Furthermore, MALAT1 knockdown reduced the levels of 15-LOX1, VEGF, the phosphorylation of STAT3 (important angiogenesis regulating factors) indicating that MALAT1 may not only control angiogenesis by ischemic stimuli but also via 15-LOX1/*STAT3* signaling pathway [61]. Moreover, MALAT1 overexpression can indirectly increase the expression of VEGFA in an OGD/R model of HBMEC. Similarly, the overexpression of MALAT1 was associated with increased angiogenesis and cell proliferation. Furthermore, it was also shown that MALAT1 acted as a ceRNA of miR-205-5p, which is able to upregulate VEGFA [62]. Consequently, these studies have confirmed that lncRNA MALAT1 is able to protect the angiogenesis function in ischemic conditions via the regulation of different miRNAs, which are henceforth able to upregulate pro-angiogenic factors (Fig. 4d).

#### LncRNA SNHG12

The role of lncRNA SNHG12 in angiogenesis after IS was determined. SNHG12 was upregulated in vitro and its overexpression was associated with increased angiogenesis and cell migration. Moreover, SNHG12 acted as a ceRNA of miR-150 and thus interfered with its target interactions. Downregulation of SNHG12 was able to enhance the tube formation (hallmark of neovascularization degree) and indirectly upregulated the level of VEGF, which was identified as a target gene of miR-150 [25]. Similarly, He et al. reported that the overexpression of miR-150 was associated with a decreased vascular density in the infarcted area, decreased tube formation, BMECs migration, and VEGF expression in in vivo analysis, confirming the previous results [23]**.** Thus, lncRNA SNHG12 also seems to have therapeutic potential in IS by regulating angiogenesis. As the number of studies are limited, further analyses are needed to confirm the therapeutic potential of MALAT1 and SNHG12 in IS (Fig. 4e).

### Excessive Oxidative Stress

Reperfusion injury is also largely attributed to the excessive production of reactive oxygen species (ROS) in ischemic tissue [63]**.** Many studies underline the association of excessive oxidative stress and lncRNAs in I/R injury. Sirtuins, which can be regulated by lncRNAs, were shown to alleviate I/R injury by protecting against cellular stress. SIRT1 in particular demonstrated beneficial effects against oxidative stress by activating FOXO1, PGC1α, and HIF2α and by inhibiting the NF-κB transcription factor [64], which makes sirtuins important contributors against I/R injury.

#### LncRNA SNHG12

The effect of lncRNA SNHG12 on I/R injury and its association with sirtuins were studied in an in vitro model. Initially, upregulated SNHG12 expression was shown in primary neuronal cells and Neuro2a (N2a) cells after OGD/R. Knockdown of SNHG12 caused inhibition of cell proliferation and increased cell apoptosis. Importantly, SNHG12 downregulated miR-199a, which inhibits cell proliferation and induces cell apoptosis; thus, SNHG12 overexpression can contribute to the I/R injury reduction. Overexpression of miR-199a or knockdown of SNHG12 inhibited the expression of *SIRT1*, suggesting that SNHG12 upregulates *SIRT1* by downregulation of miR-199a. Hence, miR-199 might be used as a biomarker to monitor the therapeutic response to SNHG12. SNHG12 can be an important regulatory factor of oxidative stress by upregulating *SIRT1* through activation of the AMPK pathway and inhibiting miR-199a in neuronal cells with I/R injury [65] (Fig. 4e). On the other hand, previous clinical study showed that miR-199 was upregulated in patients with heart failure [66]. Thus, well-designed clinical studies are needed in order to confirm the impact of SNHG12/miR-199 sponging in ischemia.

#### LncRNA H19

Another study determined the protective effect of metformin in mouse brains after MCAO, as the treatment improved neurobehavioral function and decreased infarct volume. Metformin decreased oxidative stress both in the in vitro and in vivo analysis. Importantly, metformin inhibited lncRNA H19 expression and increased the expression of miR-148a-3p*. Rock2* was determined as a target gene of miR-148a-3p, and upregulation of miR-148a-3p decreased *Rock2* expression. Therefore, the neuroprotective effect of metformin against oxidative stress injury was observed through the lncRNA H19/miR-148a-3p/*Rock2* axis [67].

Collectively, the studies showed that lncRNAs are potentially able to mediate oxidative stress injury in IS, especially via protein regulation such as sirtuins. Thus, lncRNA SNHG12 and H19 can contribute to reduced neurological impairment (Fig. 4b).

## Potentially Modifiable Factors Contributing to Recovery Following IS

The human brain possesses the capacity of self-repair after injury. Neurogenesis is a process based on the formation of neuronal cells from neural stem cells and is present in specific brain regions. Various factors such as those belonging to neurotrophic factors may enhance the process. One of the neurotrophins, brain-derived neurotrophic factor (BDNF), has a well-documented ability to promote neuroplasticity and was previously described to facilitate post-stroke rehabilitation and recovery [27, 68–74]. Recent studies indicate that IS leads to prolonged production of new striatal neurons [75]. Another factor affecting recovery after IS, post-ischemic neuroinflammation, can be influenced by modifying various pro-inflammatory factors [76]. Thus, neurological recovery can be supported by modification of neurogenesis and neuroinflammatory processes.

### Neuronal Plasticity and Neuronal Repair Mechanisms

#### LncRNA RMST

LncRNA RMST modulates neurogenesis by the regulation of neural cell fate decisions and neuronal differentiation. RMST co-regulates transcription factor SOX2 and is essential for the connection of SOX2 with its neurogenic target genes [77]. LncRNA RMST expression was found to be significantly increased in both in vitro and I/R animal models, as well with patients after IS. In vitro, RMST silencing caused decreased neuronal apoptosis and a partial reversibility of the OGD/R injury on the cell viability. In an animal model, RMST silencing caused a reduced infarction size and improved neurological function test results. Moreover, reduction of brain microgliosis and astrocytosis markers in the hippocampal region was also observed [78]. The influence of lncRNA RMST on astrocytosis and microgliosis processes is unclear. Previous studies reported that astrocytosis and microgliosis play key roles in neurological recovery, including scar formation and release of molecules promoting neuronal plasticity [79]. Nevertheless, in the acute phase of IS, astrocytosis and microgliosis have neuroprotective effects, while during the chronic phase, astrocytes may have both harmful and protective effects [80]**.**

#### LncRNA MEG3

Besides, lncRNA MEG3 plays an important role in nerve growth and neurological deficit after I/R injury. MEG3 was investigated on an animal model. In order to identify neurogenesis conditions, nerve growth factor (NGF) levels were determined, including BDNF, NGF, and basic fibroblast growth factor (bFGF). MEG3 overexpression was clinically observed as increased neurological impairment, larger infarct area, increased water content, increased blood-brain barrier permeability, neuronal apoptosis, and necrosis as well as upregulation of Wnt/B-catenin proteins, whereas MEG3 inhibitor administration resulted in opposite effects. Importantly, the treatment with MEG3 inhibitors resulted in increased BDNF, NGF, and bFGF levels. It is well known that BDNF, NGF, and bFGF are involved in neuronal repair processes, including axonal growth and proliferation of neuron progenitors [27, 28, 34, 68–70, 73, 74, 81]. Thus, the results indicate that blocking of lncRNA MEG3 expression may stimulate nerve growth and neurogenesis by increasing NGFs and may decrease neurological impairment through the Wnt signaling pathway [2]**.**

Overall, in order to identify the effective therapeutic approach, more attention should be paid to neurogenesis following IS as well as neuroprotection and reduction of post-ischemic neuronal inflammation. LncRNAs, which are able to influence the abovementioned processes, seem to be promising therapeutic approaches in IS (Table 1).

### Post-Ischemic Neuronal Inflammation

#### LncRNA MEG3

Inflammasomes are large complexes, formed in response to inflammatory stimuli, which contain various molecules such as caspases and are able to promote maturation of pro-inflammatory cytokines, i.e., interleukin-1β (IL-1β) and interleukin-18 (IL-18) [82]**.** Apart from pro-inflammatory cytokines, studies showed that AIM2 inflammasomes play an important role in ischemic brain injury. The activation of AIM2 inflammasomes can potentially induce pyroptosis and inflammation in surrounding cells, increasing the injury area [24]**.** Due to these presumptions, lncRNA MEG3-mediated modulation of AIM2 inflammasome was investigated in IS. Firstly, it was determined that MEG3 acted as a sponge for miR-485 to suppress its expression. Moreover, MEG3 and *AIM2* expressions were upregulated, whereas miR-485 was downregulated both in in vivo and in vitro analysis. Knockdown of MEG3 reduced the pyroptosis and inflammation by the upregulation of miR-485 and the downregulation of the AIM2 inflammasome signaling, whereas miR-485 inhibitor reversed the effect [26]. Similarly, Yan et al. also observed that the inhibition of MEG3 improved neurological functioning by targeting miR-21/PDCD4 axis [33] (Fig. 3h).

A handful of neuronal inflammasomes are broadly reported in literature as potential therapeutic approaches in IS [83, 84]; however, little is known about their association with lncRNAs. The AIM2 inflammasome regulated by lncRNA MEG3 is able to mediate neuronal pyroptosis, which is an inflammatory programmed cell death and is associated with membrane pore formation, cell lysis, and release of cell content. Overall, this suggests that lncRNA can potentially regulate inflammasome-mediated neuroinflammation in IS.

#### LncRNA MALAT1

The effects of lncRNA MALAT1 were also studied in post-ischemic inflammation. Silencing of MALAT1 caused a significant increase in cerebral vascular endothelial cell death and increased CASP3 activity in vitro. Similarly, silencing of MALAT1 was associated with larger infarct volumes and more serious neurological deficits in response to ischemic injury in vivo. Moreover, silencing of MALAT1 drastically increased mRNA levels of pro-apoptotic factor Bim (a member of the Bcl-2 family) and induced pro-inflammatory cytokines, such as E-selectin, monocyte chemoattractant protein (MCP-1), and interleukin-6 (IL-6) after cerebral ischemia. Additionally, it was shown that MALAT1 is a direct target for both Bim and E-selectin. Ultimately, the study recognized novel functions of MALAT1, the regulation of apoptotic, and inflammatory responses in mouse cerebral endothelium after in vitro and in vivo cerebral ischemic insults [85] (Fig. 4f).

#### LncRNA SNHG12

SNHG12 is also involved in regulation of neuroinflammation. SNHG12 was found upregulated in the in vitro model of IS. Silencing of SNHG12 increased neuronal apoptosis via enhanced expression of pro-apoptotic Bcl-2 family members. Moreover, silencing of SNHG12 also resulted in increased levels of pro-inflammatory cytokines such as IL-6 or E-selectin. Thus, SNHG12 shows anti-apoptotic and anti-inflammatory roles in ischemic conditions. Finally, the study reported that knockdown of SNHG12 is associated with a lower ratio of pAkt/Akt proteins, suggesting that Akt signaling pathway is involved in neuronal cell survival as activation of this axis reduces the survival [86]. The study shows that lncRNA SNHG12 is a promising candidate for stroke treatment in the future; however, further in vivo and human studies are needed.

#### LncRNA TUG1

Microglia play an important role in neuroinflammation. LncRNA TUG1 was upregulated in microglial cells and acted as a sponge for miR-145a-5p, negatively regulating its expression. Silencing TUG1 modified the phenotype of microglia (M1-like to M2-like) and reduced pro-inflammatory cytokines which promoted production of anti-inflammatory cytokines, thus increasing cell survival. Additionally, the OGD/R-induced activation of the NF-κB pathway was halted by the knockout of TUG1. A competitive interaction between TUG1 and miR-145a-5p was implied and it was shown that the miR-145a-5p inhibition abrogated the NF-κB inactivating effect of TUG1 knockout. Thus, the NF-κB cascade is involved in TUG1/mir-145a-5p-mediated inflammatory response. Hence, it is assumed that TUG1 can synchronize microglia and assembly of inflammatory cytokines shortly following OGD insult [87]. LncRNA TUG1 regulates microglia that are present in brain lesions. Essentially, microglia have two phenotypes, M1 which is pro-inflammatory and M2, which produces anti-inflammatory particles. TUG1 silencing promotes the differentiation to the M2 phenotype. It was shown that the M2 state is able to promote neuronal repair and regeneration via phagocytosis of harmful substances and debris in the injured area [88] (Fig. 3i).

Collectively, these studies show promising approaches for future IS treatment by targeting neuroinflammation. LncRNAs are able to decrease post-ischemic inflammation through targeting inflammasome formation and microglia phenotype change, important processes contributing to neural inflammation after IS [89]**.** Thus, all of the mentioned lncRNAs seem to be promising therapeutic approaches in IS and are therefore worthy of further clinical research (Fig. 5) (Table 1).

## Future Perspectives of lncRNAs in IS Treatment

Nowadays, available treatment methods of IS are limited and associated with possible detrimental effects such as reperfusion brain injury, and thus identification of new therapeutic approaches is urgent. Since lncRNAs are unquestionably abundant in the central nervous system and are involved in cerebral pathophysiological processes, understanding their molecular background and link with IS is essential. Numerous lncRNAs are also associated with specific neuroanatomical regions, suggesting their potentially specific functional role in the nervous system [90]. Moreover, loss and gain of function studies showed that lncRNAs may contribute to secondary damage after brain injury [91]. The abovementioned studies show that regulation of lncRNAs may exert pro-angiogenic, neuroregenerative, anti-apoptotic, and anti-inflammatory effects in injured brain tissue. Importantly, recent data demonstrates that treatment which targets non-coding RNAs or uses their molecules might be an effective approach in IS [92]. A number of studies report that miRNAs-inhibitors/mimics ameliorate IS brain injury and improve recovery and prognosis. Nevertheless, similar data about lncRNAs is limited. Current knowledge about lnRNA’s influence on IS is still not sufficient to support their use as therapeutic approaches. There is a lack of human studies and clinical trials involving lncRNAs in IS treatment. Therefore, more studies are necessary to clarify their role in this specific context as current studies showed that lncRNAs are promising approaches for ischemia.

### Advantages and Limitations of Using lncRNAs as Therapeutics in IS

One of the main advantages offered by using lncRNAs in IS treatment is their possibility to interact with miRNAs and coordinate their functions. One of the most unique regulatory roles is that lncRNAs are able to act as sponges for miRNAs. Therefore lncRNAs function as competing genes for miRNAs and inhibit/stimulate the modulatory role of miRNAs as targeted mRNAs. Moreover, as lncRNAs can regulate miRNA expression levels, it gives possible opportunities to use miRNAs as soluble biomarkers of therapeutic response to specific lncRNAs. Additionally, lncRNA may exert a more powerful effect on specific biological processes compared to miRNA. Some of the examples of structural and functional regulatory mechanisms of lncRNAs include regulation of mRNAs stability for protein synthesis, chromatin remodeling, cell cycle control, splicing regulation, targeting specific DNA sequences, as well as earlier mentioned miRNA regulation [93–95]. A number of studies confirm that the gene expression and regulation by lncRNAs are far more complex and extensive than that of miRNAs [96, 97]. Moreover, there is an increasing number of publications and novel searching methods allowing for further lncRNA investigation as well as lncRNA-miRNA interactions, including in silico prediction by using bioinformatic analysis [94, 95]. Besides, lncRNAs expression is highly specific for tissue, disease, and developmental stages [7], suggesting that therapeutic interference targeting lncRNAs might be more applicable than miRNAs targeting which are rarely specific for a single tissue [98]. On the other hand, the use of lncRNAs in clinical practice faces many limitations: (i) lack of human studies; (ii) all of those lncRNAs discussed in this review need further confirmation; (iii) individual molecules examined in patients with IS such as MALAT1 and Rian are not specific only to IS entity; (iv) although similar expression of single lncRNAs in ischemia has been confirmed independently by many researchers, describing and validation of promising therapeutics signature patterns in IS remain challenging (Fig. 5). Therefore, much work is still needed in this field. Nevertheless, the potential clinical impact is worth the investment.

## Conclusions

LncRNAs are promising targets in IS treatment, especially in the modification of reperfusion injury, which can exacerbate deficits caused by the initial ischemia [99]**.** Although the underlying mechanism of I/R injury is not fully understood, previous studies emphasized the pivotal role of mitochondrial dysfunction, excessive calcium accumulation, and glutamate excitotoxicity in neuronal apoptosis. In our review, we demonstrated that lncRNAs are able to indirectly regulate serine/threonine kinases (CaMKII, DAPK1) involved in the abovementioned neuronal cell injury pathomechanism. Thus, lncRNAs seem to have therapeutic potential on a cellular level. As it was shown, lncRNAs are also part of neuronal signaling pathways (lncRNA-miRNA-mRNA) responsible for apoptosis and IS severity. Additionally, lncRNAs are able to regulate RNA transcripts by acting as ceRNAs of miRNAs. In many studies this crosstalk could be a potential explanation of how lncRNAs control I/R injury. Moreover, in several studies, the expression rate of lncRNAs fluctuated in a specific manner and correlated with neurological deficit. This may indicate that fast lncRNA intervention may potentially limit the range of injury. Many studies have validated the crucial impact of lncRNA modification in autophagy, angiogenesis, and oxidative stress in IS. We should emphasize the importance of the lncRNAs MALAT1, H19, and SNHG12 which are involved in those processes simultaneously, thus potentially demonstrating increased therapeutic effectiveness. Furthermore, in the case of autophagy in IS, it should be noticed that autophagy has a positive effect only in the initial phases of ischemia. However, prolonged autophagy is fatal for brain tissue. Thus, lncRNAs which are able to target autophagy in IS should be highly specific for the phase of reperfusion.

Additionally, the role of lncRNAs in regulating post-ischemic inflammation should be emphasized, as many of them are able to reduce the level of pro-inflammatory cytokines. LncRNA MEG3 is able to regulate inflammasomes, a source of inflammatory molecules that are able to induce pyroptosis, an alternative neuronal cell death pathway. Moreover, lncRNA TUG1 is able to change the microglia phenotype from pro-inflammatory to anti-inflammatory, leading to enhanced neuronal repair. Last but not least, lncRNAs are involved in neurogenesis and play an important role in post-ischemic recovery. Silencing of lncRNA MEG3 increased the levels of BDNF, NGF, and bFGF, all being pivotal factors in neuronal repair processes, including axonal growth and proliferation of neuron progenitors.

Collectively, various pathological mechanisms are involved in exacerbating I/R injuries, of which the underlying processes are still not fully understood. LncRNAs can be clinically useful in IS treatment on multiple stages. In Fig. 5, miRNAs/lncRNAs, their target genes, and processes that are involved in pathophysiology of IS were summarized based on the published data. According to this network, we can conclude that lncRNA MALAT1, SNHG12, MEG3, and H19 seem to be the most promising lncRNAs as they can regulate at least three different processes.

## Data Availability

Not applicable.

## Abbreviations

15-LOX1: 15-Lipoxygenase 1
AIM2: Absent in melanoma 2
AQP4: Aquaporin 4
ATG7: Autophagy-related 7
Bcl-2: B cell lymphoma 2
bFGF: Basic fibroblast growth factor
BMECs: Brain microvascular endothelial cells
BDNF: Brain-derived neurotrophic factor
CASP3: Caspase 3
CaMKIIδ: Calcium/calmodulin-dependent protein kinase IIδ
CAMK2D: Calcium/calmodulin-dependent protein kinase II delta
CaMKII: Calcium/calmodulin-dependent protein kinase II
C2dat1: CAMK2D-associated transcript
CHRF: Cardiac hypertrophy–related factor
ceRNA: Competing endogenous RNA
DAPK1: Death-associated protein kinase 1
DNMT3B: DNA methyltransferase 3B
DUSP5-ERK1/2: Dual-specificity phosphatase 5-extracellular signal-regulated kinase ½
EC: Endothelial cells
FOXO3: Forkhead box O3
FOXO1: Forkhead box protein O1
GATA3: GATA binding protein 3
GAS5: Growth arrest-specific transcript 5
HBMECs: Human brain microvascular endothelial cells
HIF2α: Hypoxia-inducible factor 2α
IL-1: Interleukin-1
IL-18: Interleukin-18
IL-1β: Interleukin-1β
IL-6: Interleukin-6
IS: Ischemic stroke
I/R: Ischemic/reperfusion
LC3: Light chain 3 I (cytosol)
LC3 II: Light chain 3 II (membrane)
lncRNAs: Long non-coding RNAs
MALAT1: Metastasis-associated lung adenocarcinoma transcript 1
MEG3: Maternally expressed gene 3
MSCs: Mesenchymal stem cells
miRNAs miRs: MicroRNAs
MCAO: Middle cerebral artery occlusion
MAP4K4: Mitogen-activated protein kinase kinase kinase kinase 4
MCP-1: Monocyte chemoattractant protein-1
N2a: Neuro2a
NMDA: N-methyl-D-aspartate
NGF: Nerve growth factor
NF-κB: Nuclear factor kappa B
NR3C2: Nuclear receptor subfamily 3 group C member 2
OGD/R: Oxygen-glucose deprivation/reperfusion
PGC1α: Peroxisome proliferator-activated receptor gamma coactivator 1α
PDCD4: Programmed cell death protein 4
pSTAT3: Phosphorylation of signal transducer and activator of transcription 3
KCNQ1OT1: Potassium voltage-gated channel subfamily Q member1 opposite stand 1
Q PCR: Quantitative polymerase chain reaction
ROS: Reactive oxygen species
RMST: Rhabdomyosarcoma 2-associated transcript
Rock2: Rho-associated protein kinase 2
Rian: RNA imprinted and accumulated in nucleus
SIRT1: Sirtuin 1
siRNA: Small interfering RNA
SOX2: Sex-determining region Y-box 2
SOX6: Sex-determining region Y-box 6
SNHG12: Small nucleolar RNA host gene 12
TNFα: Tumor necrosis factor α
TRAF: TNF receptor-associated factor
TUG1: Taurine-upregulated gene 1
ULK2: Unc-51 like autophagy activating kinase 2
VEGFA: Vascular endothelial growth factor-A
VEGF: Vascular endothelial growth factor
Oprm1: μ-opioid receptor 1
